# Understanding Antithrombotic Agents for Rehabilitation Therapy: A Comprehensive Narrative Review

**DOI:** 10.7759/cureus.58302

**Published:** 2024-04-15

**Authors:** Shuji Matsumoto, Rintaro Ohama, Takashi Hoei, Ryuji Tojo, Toshihiro Nakamura

**Affiliations:** 1 Center for Medical Science, Ibaraki Prefectural University of Health Sciences, Ami, JPN; 2 Department of Rehabilitation Medicine, Ibaraki Prefectural University of Health Sciences Hospital, Ami, JPN; 3 Department of Rehabilitation and Physical Medicine, Kagoshima University Graduate School of Medical and Dental Sciences, Kagoshima City, JPN; 4 Department of Rehabilitation, Kagoshima University Hospital, Kagoshima City, JPN; 5 Department of Rehabilitation, Acras Central Hospital, Kagoshima City, JPN

**Keywords:** risk of bleeding, stroke, ischemic cerebrovascular disease, direct oral anticoagulant (doac), anticoagulant drugs, antiplatelet agent, rehabilitation medicine, antithrombotic agent

## Abstract

In rehabilitation medicine, attention must be paid to the medication. Among them, antithrombotic drugs are used for the initial treatment and secondary prevention of stroke, so as a basic knowledge, the pharmacological actions, characteristics, indications, and precautions for the use of antithrombotic drugs should be known. Antithrombotic agents are divided into antiplatelet agents and anticoagulants, and the appropriate antithrombotic agent is selected according to the main disease or condition. Antiplatelet agents include aspirin, clopidogrel, ticlopidine, prasugrel, ticagrelor, and cilostazol. Each antiplatelet agent has a different mechanism of action, characteristics, and indications, and should be prescribed with due consideration. Anticoagulants include heparin, synthetic Xa inhibitors, direct oral anticoagulants (DOACs), synthetic antithrombin agents, and warfarin. Knowledge of the mechanism of action, characteristics, and indications of each anticoagulant is necessary, as well as monitoring and dose adjustment. With regard to ischemic cerebrovascular disease (ICD) and antithrombotic agents, the first step is to classify cerebral infarction and to determine whether antiplatelet agents or anticoagulants should be used. Bleeding and recurrence prevention are important considerations in the selection of appropriate antithrombotic agents for the pathophysiology of ICD.

## Introduction and background

In rehabilitation medicine, it is fundamental to consider the use of medication in the assessment and treatment of function, activity, and participation according to the International Classification of Functioning, Disability and Health (ICF) [1]. About 60% of the patients in Japan's convalescent rehabilitation wards are stroke patients, and knowledge of antithrombotic agents is essential for rehabilitation treatment. Since antithrombotic agents are used in the initial treatment and prevention of recurrence (secondary prevention) of stroke, successful use of antithrombotic agents will not adversely affect rehabilitation treatment, but will improve patients' activities of daily living (ADL), thereby improving their quality of life (QOL) [2].

Due to the super-aging society and changes in lifestyle and diet, the number of patients with ischemic cardiovascular disease, non-valvular atrial fibrillation (NVAF), and deep vein thrombosis (DVT) is increasing, and the number of prescriptions for antithrombotic drugs is likely to increase every year. Recently, new antithrombotic agents and their neutralizers/antagonists have been introduced, and guidelines reflecting these new findings have been published one after another in the fields of cardiovascular and cerebrovascular disorders [3]. In daily practice, antithrombotic agents should be used in consideration of the above background and treatment guidelines [4].

This article reviews the pharmacological effects of antithrombotic agents as basic knowledge, and the precautions to be taken when prescribing them in daily clinical practice, with a focus on ischemic cerebrovascular disease (ICD).

## Review

The real world of antithrombotic agents

There are many exclusions in comparative studies of antithrombotic agents, and it is estimated that only about 30% of cases meet the evidence presented in the guidelines in real-world clinical practice. Therefore, consideration must be given to the application of antithrombotic agents in clinical practice, and real-world results of actual clinical trials play a complementary role. However, since efficacy and safety are inextricably linked for antithrombotic drugs, it should be considered that physician discretion is likely to be at work in real-world dosing.

With regard to the safety and efficacy of antithrombotic agents, enhanced antithrombotic effects increase bleeding complications. Since this is a natural consequence, mutual balance and individualized responses are required [5]. Since a wide variety of patients are involved in actual clinical practice, this individualized approach will determine safety and efficacy in the real world.

There are racial differences in the risk of thrombotic and ischemic events and the risk of hemorrhagic events, with East Asians having a higher risk of bleeding and a lower risk of thrombosis and ischemia than their Western counterparts. In fact, even with a low prothrombin time-international normalized ratio (PT-INR), the risk of warfarin-induced cerebral hemorrhage is 1.7 times higher in East Asians than in Westerners. East Asians have been shown to have a sharply increased risk of cerebral hemorrhage at edoxaban trough values >90 ng/mL, whereas Westerners have shown no association between trough value and cerebral hemorrhage [6]. In studies of prasugrel active metabolite blood levels and platelet function tests in healthy subjects, East Asians have higher active metabolite blood levels and stronger platelet function suppression than Caucasians [7]. Thus, real-world results are always required because of racial differences in drug efficacy.

Real-world outcomes of antithrombotic agents in Japan

Trends in Anticoagulant Prescribing Over Time

In the warfarin era, concerns about bleeding often led to no medication or substitution with antiplatelet agents. However, with the approval of DOACs, anticoagulant medication rates have improved over the years. The Fushimi AF Registry reported that prescriptions for NVAF cases in 2011 were 51%, 2%, and 47% for warfarin, DOACs, and no anticoagulants, respectively, and improved to 38%, 26%, and 36% in 2015, respectively [8]. In a study of 5,695 patients undergoing percutaneous coronary intervention (PCI) complicated by atrial fibrillation using personal digital cellular (PDC) data, warfarin decreased from 73.3% to 23.8% and DOAC increased from 26.7% to 76.2% between 2012 and 2017 [9]. With the advent of DOACs, anticoagulant administration rates have increased and prescribing has become DOAC-driven.

Prescription of Anticoagulants at Low Doses

With regard to the details of anticoagulant prescribing, it has been reported that substandard prescribing of low-dose anticoagulants is not uncommon. According to the XAPASS (Xarelto Post-Authorization Safety & Effectiveness Study in Japanese Patients with Atrial Fibrillation) study, a post-marketing surveillance of rivaroxaban, 35.8% of the 6,521 patients with creatinine clearance (CCr) ≥ 50 mL/min were substandard low-dose prescriptions [10]. The EXPAND (Evaluation of effectiveness and safety of Xa inhibitor for the Prevention of stroke And systemic embolism in a Nationwide cohort of Japanese patients Diagnosed as NVAF) study (5,326 patients) also showed that 30.2% of patients were non-adherent [11]. Therefore, it is estimated that in actual clinical practice in Japan, substandard low doses are selected in approximately 30% of cases. Concerns about bleeding risk, advanced age, renal dysfunction, and low body weight have been reported as the main reasons for choosing low-dose prescriptions of anticoagulants [10].

Because of the short biological half-life of DOACs, there is concern that substandard low-dose prescribing for patients with CHADS(2) scores >2 may lead to an increased risk of stroke. In fact, the aforementioned XAPASS study reported a significantly higher incidence of stroke events in patients receiving substandard low doses (2.15 vs. 1.48 events/100 patient-years, p = 0.009), even after adjusting for patient background. In the XAPASS study, there was no difference in bleeding events even at the lower dose [9]. Although it is difficult to draw conclusions, we should be cautious about prescribing easily substandard low doses.

Prescribing Anticoagulants for Patients Aged 75 Years and Older

The ANAFIE (All Nippon Atrial Fibrillation In the Elderly) study reports results for NVAF in Japan in patients over 75 years of age (mean age 81.5 years) [12]. Of the 32,275 patients, 55.6% were high-thrombotic risk cases with a CHADS(2) score of 3 or higher, and 21.1% were high-bleeding risk cases with a HAS-BLED score of 3 or higher. Of the 18,185 high thrombosis risk cases, 30.9% were also high bleeding risk cases (17.2% of the total). Overall, 7.6% of the patients were medication-free, 25.5% were prescribed warfarin, and 66.9% were prescribed DOACs [13]. Regardless of bleeding risk, DOACs were predominantly low-dose choices, with a relatively high rate of warfarin prescriptions in high-bleeding risk cases. Approximately 60% of all patients received optimal anticoagulation therapy. The overall stroke rate was 1.62% per year, and after adjusting for patient background, the 2-year prognosis for drug-free patients was a higher risk of thromboembolic events than for patients receiving anticoagulants, with no difference in risk of bleeding events [12]. In addition, patients taking DOACs had a lower risk of not only bleeding but also stroke/embolic events and all-cause mortality compared to those taking warfarin. These results suggest that anticoagulation therapy centered on DOACs is generally appropriate for the elderly.

Relatively New Antiplatelet Agents

Prasugrel and ticagrelor were newly marketed as potent platelet P2Y(12) inhibitors in 2014 and 2017, respectively. The approved dose of prasugrel is about 1/3 of the overseas dose, while ticagrelor is the same dose as the overseas dose, and prasugrel is the main newer antiplatelet agent in Japan due to bleeding complications.

Dual Antiplatelet Therapy (DAPT) After PCI

DAPT continuation at 12 months in the PRASFIT practice II post-marketing study of prasugrel (4,155 patients) was 62.2%. DAPT continuation at 12 months in the 2015-2017 PENDULUM (Platelet Reactivity in Patients with Drug Eluting Stent and Balancing Risk of Bleeding and Ischemic Event) study of PCI cases (6,267 patients) was 70-80% [13]. In addition, the 12-month DAPT continuation rate for patients who met the enrollment criteria in the STOPDAPT-2 trial but were not enrolled (2016-2017) was 73% [14]. Thus, in DAPT, physician prescribing bias in clinical practice tends to be longer-term than guideline recommendations. The trend toward longer DAPT may reflect the fact that many patients are at high risk for ischemic events such as complex PCI and acute coronary syndrome (ACS). It is also assumed that DAPT tends to be prolonged because of concerns about ischemic events rather than bleeding.

High Bleeding Risk (HBR) With Antiplatelet Drugs

The HBR definition and assessments were developed by the Bleeding Academic Research Consortium (BARC) in 2019 [15,16]. The consensus is based on real-world data, as comparative trials have excluded subsets at high bleeding risk. HBR was defined as subjects with a risk of major bleeding with BARC bleeding criteria 3 or 5 of ≥4% per year or cerebral bleeding complications of ≥1% per year on antiplatelet therapy after PCI. Based on this definition, about half of the patients in the PENDULUM trial were HBR, and HBR patients had a 3-fold higher risk of major bleeding than non-HBR patients, with a major bleeding rate of 4.1% per year [17]. These results indicate that the HBR concept is applicable in actual clinical practice in Japan. The main factors of HBR were advanced age, renal dysfunction, anemia, and anticoagulant medication.

Antiplatelet Therapy for High Bleeding Risk

The PENDULUM mono trial is a clinical study of a treatment strategy of shortened DAPT and prasugrel monotherapy for patients deemed to be at high bleeding risk. This trial showed a reduction in bleeding risk and no increase in ischemic events compared to the conventional strategy [18]. These results are consistent with a series of controlled trials and suggest that shortening the DAPT period and using a P2Y(12) inhibitor as a single agent is a valid policy that is within the discretion of the physician in real-world practice.

Types of antiplatelet agents and their mechanisms of action

Drugs commonly used as antiplatelet agents include aspirin, clopidogrel, ticlopidine, prasugrel, ticagrelor, and cilostazol. Also commonly used drugs in daily use that have antiplatelet effects include eicosapentaenoic acid (EPA), dipyridamole, and limaprost alfadex. The mechanism of action, characteristics, and indications for each drug are described below.

**Figure 1:**
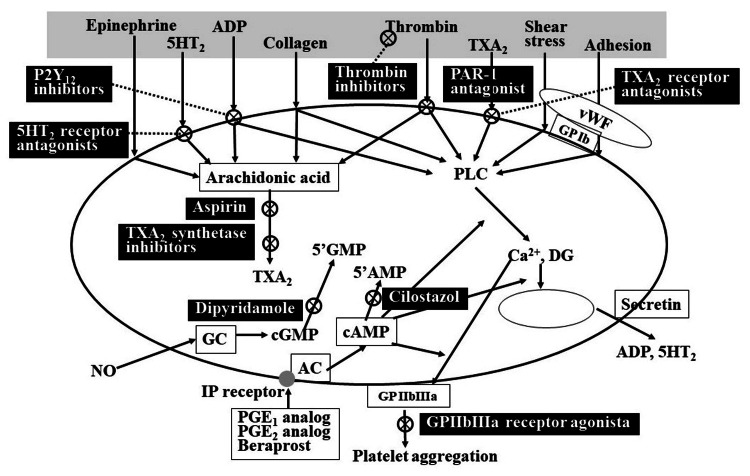
Mechanisms of platelet activation and mechanisms of action of antiplatelet agents Figure created by the author 5HT2: 5-hydroxytryptamine 2, ADP: adenosine diphosphate, TXA2: thromboxane A2, PAR-1: protease-activated receptor-1, vWF: von Willebrand factor, GP: glycoprotein, PLC: phospholipase C, DG: I, 2-diacylglycerol, GMP: guanosine monophosphate, AMP: adenosine monophosphate, GC: guanylate cyclase, AC: adenylate cyclase, NO: nitric oxide, PG: prostaglandin, IP receptor: prostacyclin receptor

When the vascular endothelium is damaged and collagen is exposed, von Willebrand factor (VWF) binds to the damaged area, and platelets adhere to the VWF via glycoprotein (GP) Ib/IX, a platelet membrane glycoprotein. Platelets aggregate with each other via fibrinogen, during which GP IIb/IIIa, a platelet membrane glycoprotein, binds to fibrinogen (primary hemostasis). The platelet thrombus thus produced is the site of a secondary hemostatic reaction. Platelets are activated in response to collagen and other external stimuli. Activated platelets not only produce the adhesion and aggregation described above but also tear off a portion of the cell wall to form microparticles (MPs), which are minute membrane endoplasmic reticulum with a diameter of 0.02 to 0.5 μm [19]. These MPs produce secondary hemostasis (coagulation activation). Activated platelets also have pro-inflammatory effects and are strongly associated with the formation of atherosclerotic lesions [19]. Antiplatelet agents produce their medicinal effects by acting on either of these platelet activation steps. The platelet activation process associated with antiplatelet agents is shown in Figure 1.

The characteristics of each antiplatelet drug

The mechanism of action, indications, and precautions for the use of the individual antiplatelet agents commonly used in Japanese medicine are described. Table 1 summarizes these drugs, as well as other drugs with antiplatelet activity.

**Table 1:** List of antiplatelet drugs and drugs with antiplatelet action and their characteristics ◎: Strong effect, 〇: Effective, △: Effect is not clear, and if there is an effect, it is negligible, X: No effect COX-1: cyclooxygenase-1, ADP: adenosine diphosphate, PDE 3: phosphodiesterase 3, TXA2: thromboxane A2, Tmax: maximum drug concentration time, T1/2: biological half-life (pharmacological half-life), SIPA: shear-induced platelet aggregation

Drug name	Mechanism of action	T_max_	T_1/2_	Duration of action	Vasodilating action	SIPA inhibition	Inhibition of vascular intima-media thickening
Aspirin	Inhibition of COX-1	4 hours	0.44 hours	7-10 days	✕	✕	△ ?
Clopidogrel	Inhibition of ADP receptors (P2Y_12_ )	1.9 hours	6.9 hours	7-10 days	✕	〇	✕
Ticlopidine	Inhibition of ADP receptors (P2Y_12_ )	2 hours	1.6 hours	8-10 days	✕	〇	✕
Prasugrel	Inhibition of ADP receptors (P2Y_12_ )	0.6 hours	0.9 hours	7-10 days	✕	〇	✕
Ticagrelor	Inhibition of ADP receptors (P2Y_12_ )	2 hours	8.7 hours	Dependent on blood concentration	✕	〇	✕
Cilostazol	Inhibition of PDE3	4 hours	18 hours	48 hours	◎	〇	〇
Eicosapentaenoic acid (EPA)	Inhibition of TxA2 synthesis	6.6 hours	60 hours	7-10 days	△	✕	△
Beraprost sodium	Stimulation of prostacyclin receptors (IP receptors)	0.56 hours	0.94 hours	Dependent on blood concentration	◎	✕	〇
Sarpogrelate	Specific antagonism of 5-HT_2_ (serotonin) receptors in platelets and vascular smooth muscle	1.2 hours	0.8 hours	Dependent on blood concentration	△	✕	△
Dipyridamole	Enhancement of adenosine action	0.5-2 hours	1.4 hours	Dependent on blood concentration	△	✕	△
Limaprost alfadex	Derivatives of prostaglandin E1	0.33 hours	0.51 hours	3 hours	〇	✕	△

Aspirin

The mechanism of action is irreversible inhibition of cyclooxygenase-1 (COX-1) in platelets and suppression of thromboxane A(2) (TXA(2)) production from platelets. Indications are inhibition of thrombus and embolus formation in angina pectoris (AP), myocardial infarction (MI), ICD, inhibition of thrombus and embolus formation after coronary artery bypass graft surgery (CABG) or post-PCI, and Kawasaki disease. As a precaution for use, COX-1 inhibition by aspirin inhibits the production of prostaglandin (PG) I2 as well as TXA(2), which may reduce blood flow to the gastric mucosa and cause peptic ulcers. Therefore, concomitant use of Proton Pump Inhibitors (PPIs) is recommended, especially in the elderly. In recent years, combination tablets of aspirin and PPI have also been used. The antiplatelet effect of aspirin is not dose-dependent, and increasing the dosage can actually decrease the antiplatelet effect (aspirin dilemma).

Clopidogrel

The mechanism of action is to inhibit adenosine 5′diphosphate (ADP) binding to the ADP receptor P2Y(12). Indications are prevention of recurrence after ICD and prevention of thrombus/embolization in ischemic heart disease for which PCI is indicated, and peripheral arterial disease. As a precaution for use, clopidogrel has a lower incidence of side effects than ticlopidine, but its pharmacokinetics are greatly influenced by the CYP2C19 gene polymorphism. Approximately 20% of Japanese patients have mutations in both alleles of the CYP2C19 gene and are poor metabolizers unable to convert clopidogrel to the active form [20]. Clopidogrel may not be fully effective in these patients.

Ticlopidine

The mechanism of action is to inhibit the binding of ADP to the ADP receptor P2Y(12). Indications include treatment of thrombi and emboli associated with vascular surgery and extracorporeal circulation and improvement of impaired blood flow disorders, improvement of various hemodynamic symptoms such as ulcers, pain, and cold sensation associated with chronic arterial occlusive disease, treatment of thrombi and emboli associated with ICD, and improvement of impaired blood flow associated with cerebral vasospasm after subarachnoid hemorrhage surgery (SAH). Precautions for use include side effects such as thrombotic thrombocytopenic purpura (TTP), severe liver injury, and agranulocytosis, especially within the first 2 weeks of ticlopidine administration. Note that ticlopidine is now less commonly used because of the introduction of clopidogrel, which has reduced these side effects.

Prasugrel

The action mechanism is to inhibit ADP binding to the ADP receptor P2Y(12). Indications are ischemic heart disease (ACS, stable AP, and old MI) for which PCI is indicated. As a precaution for use, prasugrel is metabolized by esterase in the small intestine and then the active metabolite is produced by CYP450. Prasugrel is not affected by genetic polymorphisms of CYP2C19, and individual differences in drug efficacy are unlikely to occur.

Ticagrelor

The mechanism of action is selective and reversible antagonism of the ADP receptor P2Y(12), binding to a site distinct from the ADP binding site and inhibiting signaling from the P2Y(12) receptor. Indications are in cases of old MI with a particularly high risk of developing atherothrombosis and in cases of ACS for which PCI is indicated. In terms of precautions for use, ticagrelor is a direct and competitive inhibitor of P2Y(12). The onset of the effect is rapid due to the active form, and the binding to the receptor is reversible, so the effect disappears promptly after drug withdrawal.

Cilostazol

By inhibiting phosphodiesterase (PDE) 3, cilostazol inhibits the degradation of cyclic adenosine monophosphate (cAMP) to 5'AMP, the mechanism of action. As a result, cilostazol increases the concentration of cAMP in platelets, inhibiting the increase in platelet Ca concentration and exerting an antiplatelet effect. Indications include improvement of ischemic symptoms such as ulcers, pain, and cold sensation based on chronic arteriosclerosis obliterans, and prevention of recurrence after cerebral infarction (excluding cardiogenic cerebral embolism). In terms of precautions for use, since PDE3 also exists in vascular smooth muscle, cilostazol has both vasodilating and vasoactive effects [21], but improvement of cerebral blood flow may cause a feeling of heaviness in the head, making it difficult to continue treatment in some cases. Cilostazol also increases cAMP in cardiac myocytes, resulting in positive alteration and positive potentiation, often causing tachycardia, although bradycardia may improve in some patients with sinus failure.

Antidepressant and antiplatelet effects

The use of selective serotonin reuptake inhibitors (SSRIs) [22] and serotonin noradrenaline reuptake inhibitors (SNRIs), which are frequently used as antidepressants in recent years, may pose a risk of bleeding. This is thought to be because serotonin release from platelets at the site of bleeding is inhibited during SSRI and SNRI administration, which also inhibits platelet aggregation. When combined with antiplatelet agents, bleeding risk should be further monitored [23]. In fact, there have been many reports of purpura, gastrointestinal bleeding, and epistaxis with the use of SSRIs [24]. Whether the use of SSRIs in combination with warfarin or DOACs increases the risk of bleeding has yet to be evaluated [25,26].

Severe acute respiratory syndrome coronavirus 2 (SARS-CoV-2) practice and antiplatelet agents

For SARS-CoV-2 patients who are obese, immobile, or have a D-dimer greater than three to four times the upper limit of normal, anti-coagulation therapy with heparin or other drugs is recommended. Although there is no mention of antiplatelet agents for SARS-CoV-2 in the Japanese practice guidelines, it has been reported from overseas that the mortality rate is lower in patients using aspirin (odds ratio: 0.50, 95% CI: 0.36-0.69) than in those not using aspirin (odds ratio: 0.50, 95% CI: 0.36-0.69) [27]. On the other hand, there are reports that aspirin treatment did not improve mortality [28], and this is an issue for future study. The pros and cons of aspirin may depend on the timing of administration, how it is used in combination with other antithrombotic therapies, and background disease.

Antiplatelet agents that also have vasodilating effects

Among the antiplatelet agents discussed in this paper, those with clear vasodilating properties are cilostazol and beraprost sodium. These agents are therefore used for chronic arterial occlusive disease and arteriosclerosis obliterans. However, because vasodilation is associated with side effects (e.g., lightheadedness, tachycardia, etc.), it is advisable to start with small doses and titrate up gradually, rather than administering a full dose from the beginning. Peak blood concentrations and half-lives are shown in Table 1. Both the effects and side effects of antiplatelet agents are often seen at peak blood concentrations. Antiplatelet agents with vasodilating effects are also effective in Raynaud's phenomenon, but if Raynaud's symptoms have a time-of-day characteristic, changing the time of administration with an awareness of the blood concentration trend may have a dramatic effect.

Types, mechanisms of action, and characteristics of anticoagulants

Anticoagulants used in Japan can be classified as shown in Table 2. Each is outlined below.

**Table 2:** List of anticoagulants and their characteristics ADP: adenosine diphosphate, Tmax: maximum drug concentration time, T1/2: biological half-life (pharmacological half-life), P-gp: P-glycoprotein

Drug name	Mechanism of action	T_max_	T_1/2_	Peak blood concentration time	Protein binding rate	Renal excretion	Bioavailability	Potential for drug interactions
Unfractionated heparin	Inhibition of Xa and IIa	Dependent on dose and method of administration	45-60 minutes	Dependent on dose and method of administration	Not known	90%	30%	Anticoagulants/Urokinase/Alteplase/Aspirin/Ticlopidine
Low molecular weight heparin	Inhibition of Xa and Iia (Xa > IIa)	Dependent on dose and method of administration	2-4 hours	Dependent on dose and method of administration (20 hours)	Not known	90%	100%	Anticoagulants/Urokinase/Alteplase/Aspirin/Ticlopidine
Heparinoid: danaparoid	Inhibition of Xa and Iia (Xa >> IIa)	3 hours	20 hours	30 minutes	93%	90%	100%	Warfarin/Aspirin/Dipyridamole/Urokinase
Synthetic Xa inhibitor	Inhibition of ADP receptors (P2Y_12_ )	2 hours	14-17 hours	2 hours	97-99%	80%	101%	Anticoagulants/Aspirin/Alteplase/Ticlopidine
Apixaban	Inhibition of Xa	3-4 hours	8-15 hours	1-4 hours	87%	25%	50%	CYP3A/P-gp inhibitor
Rivaroxaban	Inhibition of Xa	2 hours	5-9 hours	2-4 hours	92-95%	33%	66-100%	CYP3A4/P-gp inhibitor
Edoxaban	Inhibition of Xa	1-2 hours	9-10 hours	1-2 hours	40-59%	35-39%	50-60%	CYP3A/P-gp inhibitor
Dabigatran	Inhibition of IIa	1.25-2 hours	12-17 hours	2 hours	35%	80%	3-7%	P-gp inhibitor
Synthetic antithrombin agent	Inhibition of IIa	40-50 minutes	15-30 minutes	15-20 minutes	54%	23%	100%	Heparin/Warfarin/Aspirin/Piroxicam
Coumarin anticoagulant, warfarin	Inhibition of production of II, VII, IX, and X factors	120 hours	36-48 hours	1-12 hours	99%	0%	100%	CYP2C9

Unfractionated Heparin and Low Molecular Weight Heparin

Heparin is a highly sulfated form of heparan sulfate, and its basic structure is a linear polysaccharide consisting of tens to hundreds of polymerized units of disaccharides, uronic acid, and glucosamine [29]. Unfractionated heparin has varying degrees of polymerization and molecular weights from 3 to 30 kDa. Low molecular weight heparin, on the other hand, is a modified version of unfractionated heparin with a molecular weight of about 1/3 of that of heparin. Heparin itself has no anticoagulant effect but binds to antithrombin by a specific pentasaccharide sequence that can bind to antithrombin and inhibit coagulation by enhancing the activity of antithrombin (IIa) and blood coagulation factor X by several hundred to several thousand times over that of thrombin alone [30].

Because heparins are predominantly renally metabolized, dose reduction is necessary for patients with renal impairment [31]. Unfractionated heparin has a short biological half-life of 45-60 minutes, while low molecular weight heparin has a relatively long biological half-life of 2-4 hours. The anticoagulant effect can be monitored by the activated partial thromboplastin time (APTT) with unfractionated heparin, whereas low molecular weight heparin hardly prolongs the APTT and therefore its coagulant effect cannot be monitored. Activated coagulation time (ACT) is used for monitoring endovascular procedures such as PCI and extracorporeal membrane oxygenation (ECMO) because it is simple, quick, and suitable for monitoring high-dose heparin [32]. Unfractionated heparin is used for a variety of medical conditions, including disseminated intravascular coagulation (DIC), but most low molecular weight heparins are indicated for extracorporeal circulation, such as hemodialysis. Dalteparin is also indicated for DIC, and enoxaparin is indicated for the prevention of postoperative DVT (Table 2). Protamine sulfate, used as a neutralizer of heparin, has a positive charge and forms a stable complex with unfractionated heparin, which has a strong negative charge, thereby inhibiting anticoagulation. Protamine sulfate is therefore less effective against low molecular weight heparin and ineffective against danaparoid and fondaparinux.

Unfractionated, low molecular weight heparin forms a complex with platelet factor 4 (PF4), which is the antigen for the production of anti-platelet factor 4/heparin complex antibodies. Some of these antibodies activate platelets and monocytes, causing overproduction of thrombin, resulting in thrombocytopenia and arteriovenous thromboembolism. Usually develops 5-14 days after heparin administration, with a platelet count of about 60,000/µL, but rarely results in bleeding and arteriovenous thromboembolism, including pulmonary embolism, MI, cerebral infarction, and cerebral venous thrombosis. Heparin-induced thrombocytopenia (HIT) is estimated to occur in about 0.1% to 1% of heparin-treated patients [33]. The diagnosis of HIT is assessed by 4T's scoring (thrombocytopenia, timing of platelet count fall, thrombosis, and other causes of thrombocytopenia) and antibody measurement. Treatment of HIT includes immediate discontinuation of heparin and administration of argatroban.

Heparinoid: Danaparoid

Extracted from the small intestine of pigs and stripped of heparin and its fragments, it has an anti-Xa/thrombin activity ratio of 22:1, even more selective for factor Xa inhibition than low molecular weight heparins (2-5:1 activation) [34]. The binding of danaparoid to antithrombin is reversible and does not inhibit antithrombin binding to vascular endothelium, a physiological anti-DIC response, and thus does not inhibit anti-inflammatory activity. Danaparoid is renally excretory and has a very long half-life of 20 hours.

Synthetic Xa Inhibitor: Fondaparinux

Fondaparinux is a chemically synthesized pentasaccharide that binds specifically to antithrombin and has an anti-Xa/thrombin activity ratio of 7,400:1, almost exclusively Xa inhibition (Table 2) [35]. Fondaparinux does not bind to endothelial cells or plasma proteins. Fondaparinux has a long half-life of 14-17 hours and there is no way to monitor it.

DOAC

DOACs are oral anticoagulants that can directly inhibit thrombin and factor Xa, with a biological half-life of almost half a day. DOACs are available in once- or twice-daily dosing regimens and are used for the prevention of embolism from NVAF and venous thromboembolism [36]. Dabigatran, a thrombin inhibitor, is not indicated for venous thromboembolism (Table 2). Dabigatran often results in mild prolongation of the APTT, but there is no precise monitoring with respect to DOAC that reflects the drug's efficacy. Dabigatran has a specific neutralizer, idarucizumab, while apixaban, rivaroxaban, and edoxaban have a specific neutralizer, andexanet alfa.

Synthetic Antithrombin Agents: Argatroban

Argatroban is a synthetic antithrombin drug developed in Japan, a compound with a molecular weight of 530 with an arginine backbone. It directly and selectively inhibits thrombin without depending on antithrombin. Argatroban reaches a steady state within 1-3 hours of administration, and unlike heparin, it also acts on fibrin-bound thrombin. Argatroban is indicated for the treatment of acute cerebral thrombosis (excluding lacunar infarction) within 48 hours of onset [37], chronic arterial occlusive disease (Buerger's disease, arteriosclerosis obliterans), congenital antithrombin deficiency and HIT (Table 2).

Coumarin Anticoagulant: Warfarin

**Figure 2:**
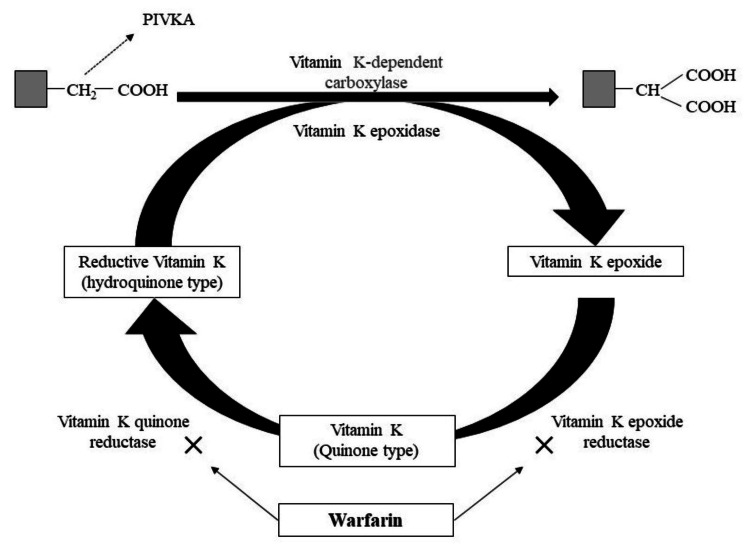
Action mechanism of warfarin Figure created by the author PIVKA: protein induced by vitamin K absence or antagonists

Warfarin exerts its anticoagulant effect by irreversibly inhibiting the enzymatic activity of both vitamin K epoxidoreductase (VKOR) and vitamin K quinone reductase in the liver, leaving the vitamin K-dependent coagulation factor as the incomplete coagulation factor protein induced by vitamin K absence or antagonists (PIVKA) without the gamma carboxyglutamic acid (Gla) residue action (Figure 2).

Warfarin is completely absorbed from the upper gastrointestinal tract after oral ingestion, reaching maximum blood concentrations about 1 to 2 hours after oral administration. In the blood, warfarin is mostly bound to albumin, which is metabolized in the liver with a half-life of approximately 40 hours (Table 3). The pharmacologically active S-warfarin is metabolized mainly by CYP2C9, a cytochrome P450 in the liver, and genetic polymorphisms of CYP2C9 and VKOR produce individual differences in drug efficacy.

**Table 3:** Characteristics of warfarin and DOACs DOAC: direct oral anticoagulant, CCr: creatinine clearance, HIV: Human Immunodeficiency Virus, APTT: activated partial thromboplastin time, PT-INR: prothrombin time-international normalized ratio

Drug name	Target factors	Number of doses	Monitoring	Contraindications in CCr	Criteria for dose reduction	Concomitant use contraindicated
Apixaban	Factor Xa	Twice a day	None	CCr less than 15mL/min	Over 80 years of age, Serum Cr > 1.5, Body weight less than 60 kg	None
Rivaroxaban	Factor Xa	Once a day	None	CCr less than 15mL/min	CCr 15-49mL/min, clarithromycin, erythromycin, fluconazole	HIV protease inhibitors/itraconazole
Edoxaban	Factor Xa	Once a day	None	CCr less than 15mL/min	CCr 15-49mL/min, P-glycoprotein inhibitor	None
Dabigatran	Thrombin (Factor IIa)	Twice a day	APTT	CCr less than 30mL/min	CCr 30-49mL/min, P-glycoprotein inhibitor, 70 years old and over, history of gastrointestinal bleeding	Itraconazole
Warfarin	Vitamin K-dependent coagulation factor	Once a day	PT-INR	None	Vitamin K2 for osteoporosis/iguratimod/miconazole	Vitamin K2 for osteoporosis/iguratimod /Miconazole

Vitamin K-dependent coagulation factors include prothrombin (Factor II), Factor VII, Factor IX, and Factor X. Of these, Factor VII has the shortest biological half-life and is monitored by PT and evaluated by the international standard ratio PT-INR [38]. However, the PT-INR does not always accurately reflect the effect of treatment, since anticoagulation is largely due to a decrease in Factor X and Factor II. Administration of vitamin K can neutralize the effects of warfarin, but it takes 12-24 hours for the PT-INR to recover. If immediate neutralization is needed, coagulation factor replacement with blood products is necessary, and vitamin K-dependent coagulation factors can be rapidly replenished by prothrombin complex preparations. Warfarin is used to treat and prevent thromboembolism (venous thromboembolism, MI, pulmonary embolism, cerebral embolism, etc.), but is contraindicated in pregnant women due to teratogenicity. It has strong dietary and drug interactions and requires regular PT-INR monitoring and dose adjustment.

ICD and antithrombotic agents in Japan

Epidemiology of ICD

The total number of strokes in Japan exceeds 1.1 million, with approximately 300,000 new strokes occurring each year. ICDs account for 3/4 of all strokes that occur. Most ICDs develop on the basis of risk factors for atherosclerosis, such as hypertension, diabetes, dyslipidemia, and smoking, or in association with NVAF, which is common in the elderly. In Japan, a super-aging society, there are many cases with risk factors for atherosclerosis and NVAF, and there is a demand to prevent the onset and recurrence of ICDs by managing each risk factor and appropriate antithrombotic therapy.

Classification of ICD

It is important to properly classify cerebral infarction, as the subsequent treatment plan depends on the pathology. The Trials of Org 10172 in Acute Stroke Treatment (TOAST) classification, currently widely used to classify cerebral infarction, is characterized by its clear and simple criteria and includes three types of infarction. Based on the pathologic mechanism, the three major types are (1) atherothrombotic cerebral infarction, (2) cardiogenic cerebral embolism, (3) lacunar infarction, (4) cerebral infarction due to other causes, and (5) cerebral infarction of unknown cause [39].

Venous Thrombolysis (Fibrinolysis)

For acute cerebral infarction in the early stage of onset, intravenous thrombolysis (recombinant tissue-type plasminogen activator: rt-PA) using alteplase (0.6 mg/kg) should be considered if the onset is within 4.5 hours. Patients with ICDs in all clinical categories are eligible for rt-PA therapy. Alteplase administration has been shown to slightly increase the incidence of symptomatic intracranial hemorrhage, but not mortality, and to significantly increase the number of patients with a good outcome (modified Rankin Scale 0 to 1) 3 to 6 months after onset. The earlier treatment is initiated, even within 4.5 hours of onset, the better outcome can be expected. After selecting patients to be considered for alteplase administration based on time of onset, check for off-label (contraindications) (Table 4) and prudent administration. The decision to administer alteplase is based on time course, history, clinical, blood, and imaging findings. If even one off-label item is present, do not administer alteplase.

**Table 4:** Off-label items for venous thrombolytic therapy SBP: systolic blood pressure, DBP: diastolic blood pressure, PT-INR: prothrombin time-international normalized ratio, APTT: activated partial thromboplastin time, CT: computed tomography, MRI: magnetic resonance imaging

Off-label (contraindicated)
Previous medical history
Non-traumatic intracranial hemorrhage
- Cerebral infarction within 1 month
- Serious head and spinal cord trauma or surgery within 3 months
- Gastrointestinal or urinary tract bleeding within 21 days
- Major surgery or serious trauma other than head injury within 14 days
Clinical findings
- Complications of subarachnoid hemorrhage or acute aortic dissection
- Complicated bleeding (intracranial, gastrointestinal, urinary tract, retroperitoneal, hemoptysis)
- SBP ≥ 185 mmHg or DBP ≥ 110 mmHg after antihypertensive therapy
- Severe hepatic disorder, acute pancreatitis
- Infective endocarditis
Blood findings
- Abnormal blood glucose (corrected blood glucose <50 mg/dL or >400 mg/dL)
- Platelet count ≤ 100,000/mm3
- Patients on anticoagulant therapy or with abnormal coagulation, PT-INR >1.7, APTT >1.5
CT/MRI findings
- Extensive early ischemic changes, evidence of compression (midline shift)

Antiplatelet therapy

Antiplatelet Therapy in the Acute Phase

In the acute phase, antiplatelet agents are administered to prevent recurrence in noncardiogenic stroke. For cerebral infarction within 48 hours of onset, oral aspirin 160-300 mg/day is effective in improving patient outcomes [3]. In the acute phase, it is also recommended to administer a combination of two antiplatelet agents instead of a single agent. In the CHANCE (Clopidogrel in High-Risk Patients With Acute Non-Disabling Cerebrovascular Events) study, the 21-day combination of aspirin and clopidogrel (and clopidogrel alone thereafter) demonstrated higher efficacy and safety at 3 months compared to aspirin alone [40]. While DAPT strongly reduces recurrent stroke, there is concern about the increased risk of bleeding, but the CHANCE study did not show an increase in serious bleeding events compared to monotherapy. The optimal duration of DAPT administration was examined in a meta-analysis that included the CHANCE study and showed that the combination of DAPT within 1 month was effective in preventing recurrence without increasing cerebral hemorrhage or major bleeding [41]. In clinical practice, aspirin 200 mg and clopidogrel 300 mg are often administered initially, followed by aspirin 100-200 mg/day and clopidogrel 75 mg/day thereafter. After 2-3 weeks of hospitalization in an acute care facility, the patient is switched to clopidogrel or aspirin alone when discharged home or transferred to a hospital.

Cilostazol 200 mg/day has shown similar efficacy and safety as aspirin, and DAPT with aspirin can be considered. Ozagrel sodium 160 mg/day is available as an intravenous drug, but data on DAPT are lacking. Triple therapy with antiplatelet agents is not recommended as initial treatment in the acute setting because it does not prevent recurrent stroke and significantly increases major bleeding [3].

Antiplatelet Therapy in the Chronic Phase

In noncardiogenic cerebral embolism, antiplatelet agents should be administered in the chronic phase following acute therapy. Aspirin 75-150 mg/day, clopidogrel 75 mg/day, and cilostazol 200 mg/day are effective in preventing recurrent noncardiogenic cerebral embolism [3]. Antithrombotic medications may be discontinued during surgery or laboratory tests, depending on their bleeding risk. Antithrombotic medications should be continued for minor surgeries and procedures in which bleeding can be easily managed.

Anticoagulation therapy

Anticoagulation Therapy in the Acute Phase

In cardiogenic cerebral embolism, anticoagulants are chosen to inhibit thrombus formation in the left atrium or left ventricle. Early introduction of anticoagulation is desirable in the acute phase because of the high recurrence rate, but on the other hand, there is concern that melting of the embolus elements and reopening of the vessel may contribute to hemorrhagic infarction. This risk is particularly high with heparin and warfarin. On the other hand, DOACs have a substantially lower incidence of hemorrhagic stroke than heparin or warfarin. In cases of acute cerebral infarction associated with NVAF, DOACs are often introduced within 3 days in mild cases and within 1 week in severe cases, depending on the size of the infarcted lesion and the severity of symptoms [42,43]. For cerebral infarction with an unknown embolic source, treatment is often initiated with 10,000 units of heparin/day and switched to warfarin or antiplatelet agents, depending on the final diagnosis. Argatroban is used in the acute phase of cerebral thrombosis within 48 hours of onset, as mentioned above.

Anticoagulation Therapy in the Chronic Phase

In the chronic phase of cardiogenic cerebral embolism, anticoagulation with DOACs and warfarin is given. In the prevention of recurrent stroke or transit ischemic attack (TIA) associated with NVAF, DOACs should be considered first, followed by warfarin if DOACs cannot be administered [44]. In Japan, there are four types of DOACs, and their dosage and administration are shown in Table 3. The decision to administer each DOAC or warfarin should be made through a collaborative decision-making approach based on information about each drug from the medical staff and discussions based on the patient's condition and needs.

For minor bleeding, anticoagulants should be continued and patients should be monitored. For moderate to severe bleeding, consider appropriate hemostatic measures, withdrawal of medication, or administration of a neutralizing agent while maintaining circulatory stability. In severe bleeding during warfarin therapy, prothrombin complex preparations and 10 mg of vitamin K are first considered. Fresh-frozen plasma is not as effective. The dosage of prothrombin complexes should be based on PT-INR and body weight [44]. Neutralizers have been developed for the development of life-threatening or difficult-to-hemostop bleeding during DOAC administration [43].

## Conclusions

Antithrombotic agents are broadly classified into antiplatelet agents and anticoagulants, and the appropriate antithrombotic agents should be selected based on the pathophysiology of the patient, such as ischemic cerebrovascular disease, post-percutaneous coronary intervention, or atrial fibrillation. In addition, the mechanism of action, characteristics, and indications of antiplatelet and anticoagulant drugs commonly used in daily clinical practice should be known in order to provide rehabilitation therapy. In clinical practice, anticoagulants tend to lean toward lower doses due to concerns about bleeding, and conversely, dual antiplatelet therapy after percutaneous coronary intervention tends to lean toward long-term dual antiplatelet therapy due to concerns about thrombotic events. Correct risk assessment and dosing are key to maintaining efficacy and safety. To avoid hemorrhagic complications associated with antithrombotic agents, especially major bleeding, it is important to manage the risk of intracranial bleeding (hypertension, high blood sugar, smoking, excessive alcohol consumption, and head bruising). If gastrointestinal bleeding is a concern, Helicobacter pylori eradication and fecal occult blood testing should be performed as needed, and concomitant use of antithrombotic agents should be avoided as much as possible.
